# Factors Influencing Implementation of eHealth Technologies to Support Informal Dementia Care: Umbrella Review

**DOI:** 10.2196/30841

**Published:** 2021-10-08

**Authors:** Sofia Bastoni, Christian Wrede, Marcia Cristina da Silva, Robbert Sanderman, Andrea Gaggioli, Annemarie Braakman-Jansen, Lisette van Gemert-Pijnen

**Affiliations:** 1 Department of Psychology, Health & Technology Centre for eHealth and Wellbeing Research University of Twente Enschede Netherlands; 2 Department of Health Psychology University Medical Center Groningen Groningen Netherlands; 3 Department of Psychology Universitá Cattolica del Sacro Cuore Milan Italy; 4 Applied Technology for Neuro-Psychology Lab Istituto Auxologico Italiano Milan Italy

**Keywords:** eHealth, assistive technologies, dementia, informal care, home care, implementation

## Abstract

**Background:**

The worldwide increase in community-dwelling people with dementia underscores the need for innovative eHealth technologies that aim to provide support to both patients and their informal caregivers in the home setting. However, sustainable implementation of eHealth technologies within this target group can be difficult.

**Objective:**

The goal of this study was to gain a thorough understanding of why it is often difficult to implement eHealth technologies in practice, even though numerous technologies are designed to support people with dementia and their informal caregivers at home. In particular, our study aimed to (1) provide an overview of technologies that have been used and studied in the context of informal dementia care and (2) explore factors influencing the implementation of these technologies.

**Methods:**

Following an umbrella review design, five different databases were searched (PubMed, PsycINFO, Medline, Scopus, and Cochrane) for (systematic) reviews. Among 2205 reviews retrieved, 21 were included in our analysis based on our screening and selection procedure. A combination of deductive and inductive thematic analyses was performed, using the Nonadoption, Abandonment, Scale-Up, Spread, and Sustainability (NASSS) framework for organizing the findings.

**Results:**

We identified technologies designed to be used “by informal caregivers,” “by people with dementia,” and “with people with dementia.” Within those groups, most of the represented technologies included, respectively: (i) devices for in-home monitoring of lifestyle, health, and safety; (ii) technologies for supporting memory, orientation, and day structure; and (iii) technologies to facilitate communication between the informal caregiver and person with dementia. Most of the identified factors influencing implementation related to the condition of dementia, characteristics of the technology, expected/perceived value of users, and characteristics of the informal caregiver. Considerably less information has been reported on factors related to the implementing organization and technology supplier, wider institutional and sociocultural context of policy and regulations, and continued adaptation of technology over time.

**Conclusions:**

Our study offers a comprehensive overview of eHealth technologies in the context of informal dementia care and contributes to gaining a better understanding of a broad range of factors influencing their implementation. Our results uncovered a knowledge gap regarding success factors for implementation related to the organizational and broader context and continuous adaptation over the long term. Although future research is needed, the current findings can help researchers and stakeholders in improving the development and implementation of eHealth technologies to support informal dementia care.

## Introduction

### Background

Dementia affects more than 50 million people worldwide [1] and this number is expected to triple by 2050 [2]. To reduce the tension between an increasing demand for care and the growing shortage in residential care capacity [3], many countries have shifted their attention toward deinstitutionalization and to support people with dementia to live at home for as long as possible [4,5]. Although extended independent living is preferred by most people with dementia [6], this also puts more pressure on their informal caregivers [7] such as spouses, children, or other relatives providing unpaid care at home. The volume of informal care has already been relatively large in most European countries, making up most of the care received by those aged 50 years or older [8,9]. Informal caregivers of people with dementia can feel heavily burdened by their care responsibilities, often resulting in stress-related symptoms such as anxiety [10], mood, or sleep disorders [11]. The strain on informal caregivers and people with dementia has become even more present in light of the COVID-19 pandemic as routine professional care services were postponed or decreased [12], inevitably causing a greater reliance on home care as one pillar of the health care system [13].

The increasing need for support of both patients and their informal caregivers in the home setting has led to innovative solutions, including those from the field of eHealth technology [14]. In the Netherlands, the National Dementia Strategy 2021-2030 [15] promotes the utilization and further development of eHealth technologies to support both patients and caregivers. The goals of these technologies within dementia care are diverse. A systematic review performed by Ienca et al [16] distinguished several purposes of (smart) technologies for dementia care, including assistance for activities of daily living, cognitive and emotional assistance, health and behavioral monitoring, fostering social interaction, and remote communication and emergency systems. In this review, we consider eHealth as “the use of technology to support health, well-being, and healthcare” [17]. This rather inclusive and broad definition covers all (intelligent) assistive technologies and technology-based interventions that can be used to support people with dementia and their informal caregivers in the home setting.

Despite their promising potential and position within recent policy, the use of eHealth technologies in dementia care is unfortunately still limited [18]. Low adoption rates may signal problems during implementation. In fact, the sustainable implementation of eHealth technologies aimed at providing support in home-based settings is frequently unsuccessful in daily practice [19,20], resulting in these technologies falling into the “valley of death” [21] after the research projects have ended. This raises a question about what exactly facilitates or hinders the successful implementation of a broad range of eHealth technologies in the informal dementia care context, which so far has been given little attention in research [14].

A previous scoping review performed by Guisado-Fernández et al [22] investigated factors influencing the adoption of “smart health technologies” [22] for people with dementia and their informal caregivers. Their review identified attitudinal aspects, ethical issues, design-related issues, and dementia-related challenges as playing key roles. Another review by Christie et al [14] focused on the implementation of digital interventions for informal caregivers of people with dementia. Several determinants were identified, including perceived data security, psychological characteristics of caregivers, care policy, or financial constraints. Both studies illustrate simultaneously that knowledge about factors influencing the implementation of eHealth technologies in informal dementia care is currently fragmented across different studies in the literature. Previous reviews tended to either focus primarily on a specific part or outcome of implementation (eg, adoption or acceptance) [22,23] or zoomed in on a specific type of technology [14,24]. This makes it difficult to obtain a complete overview of supportive eHealth technologies in the context of informal dementia care and what factors facilitate or impede their implementation.

To the best of our knowledge, no review yet exists that aims to summarize the influential factors across the whole spectrum of implementation and related to a broad range of technologies studied in the specific context of informal dementia care. For such a review to deliver beneficial and complete results, we consider the guidance of a holistic view on implementation as essential. Numerous implementation frameworks exist that view implementation as a postdesign phase [25-27], although it is currently recommended to target aspects of implementation at an early stage of development [17]. By contrast, the Nonadoption, Abandonment, Scale-Up, Spread, and Sustainability (NASSS) framework [28] considers the implementation process in a multilevel and comprehensive fashion, which encompasses the influences on the adoption, nonadoption, abandonment, spread, scale-up, and sustainability of eHealth technologies. This evidence-based and theory-informed framework includes both adoption and acceptance from the viewpoint of the stakeholders but also considers aspects of implementation that relate to the wider context [28]. Because of its holistic view on implementation, the NASSS framework was used as a general guide in our study.

### Aims of the Study

The aim of this review was to gain a more complete understanding of why it is often difficult to implement and integrate eHealth technologies in everyday life despite the numerous technologies that have been studied and designed to support people with dementia and their informal caregivers at home. In particular, the complementary study aims were to (1) provide an overview of the types of technologies that have been used and studied in the context of informal dementia care and (2) explore the factors influencing the implementation of those technologies.

The findings of this review are expected to be useful in determining directions for future research, and to help researchers and stakeholders in improving the development and implementation of eHealth technologies to support informal dementia care.

## Methods

### Approach

We used an umbrella review design to create an overview of the state of the art of technology for supporting informal dementia care and to identify determinants for its implementation. The methodology of an umbrella review can best be described as a systematic review of reviews, meaning that reviews are used as the analytical unit of the umbrella review [29]. Umbrella reviews are particularly fitting in a broad field of work and aim to provide a summary of the highest-quality studies into the state of the art of a certain domain. With this approach, gaps in the literature are highlighted and principal findings are presented in a tabular and concise manner that can be used in practice [29].

### Search Strategy

A systematic literature search of five databases (PubMed, PsycINFO, Medline, Scopus, and Cochrane) was performed in June 2020. The aims of the search were to identify reviews that describe (i) technologies that are in use or have been studied to support informal dementia care and (ii) information related to the implementation of those technologies. The search string included terms pertaining to four main categories of keywords: (i) eHealth, (ii) Implementation, (iii) Informal Care, and (iv) Dementia. To build the search string, thesaurus and nonthesaurus terms were used. The search string was then adapted to each database and approved by a library consultant at the University of Twente. In general, the four categories were separated by AND, and the single terms inside of each category were separated by OR. The complete search string and database adaptations can be found in Multimedia Appendix 1.

### Inclusion and Exclusion Criteria

Included studies needed to be reviews containing information related to the implementation of eHealth technologies for people with dementia and/or their informal caregivers. Studies in English, German, Dutch, Italian, and Portuguese languages were searched. Nonreview articles such as primary studies, randomized controlled trials, books, dissertations, and grey literature were excluded, as recommended for umbrella reviews [30]. Studies that also included non-eHealth interventions or other types of diseases were excluded. Furthermore, studies published before 2010 were excluded as it was believed that

these would not add relevant information since those technologies are likely to have become outdated.

### Data Extraction

During title and abstract screening, each paper was independently evaluated by at least two reviewers (SB, MS, or CW) and conflicts were solved through discussion. For full-text screening, the same procedure was followed, but consensus was reached through unanimity. Studies that fit all inclusion criteria were examined using a data extraction form on Covidence. The form was used to extract data about study details (eg, title, year, author, type of review, number of included studies), information about included technologies (eg, technology types, purposes, and primary user groups), and any statements related to implementation, including potential barriers and facilitators. Each paper was randomly assigned to at least two of the three reviewers (SB, CW, MS) who performed the data extraction independently from each other. Subsequently, completed data extraction forms for each paper were reviewed and adapted until consensus among all reviewers was reached.

### Data Analysis

A qualitative thematic analysis was performed on the extracted data using Atlas.Ti9.

The NASSS framework was used to categorize the data. This framework consists of seven domains and can be used to evaluate the success of implementation of a health technology retrospectively and prospectively. Relevant fragments were selected and categorized into (1) one of the seven domains of the NASSS framework or (2) classified as miscellaneous information. Subsequently, selected fragments were further categorized inductively into overarching themes. To minimize single-researcher bias, the coded papers were checked independently by a second researcher. The final coding scheme was developed and defined based on consensus among the three researchers (SB, CW, MS).

## Results

### Characteristics of the Included Studies

**Figure 1:**
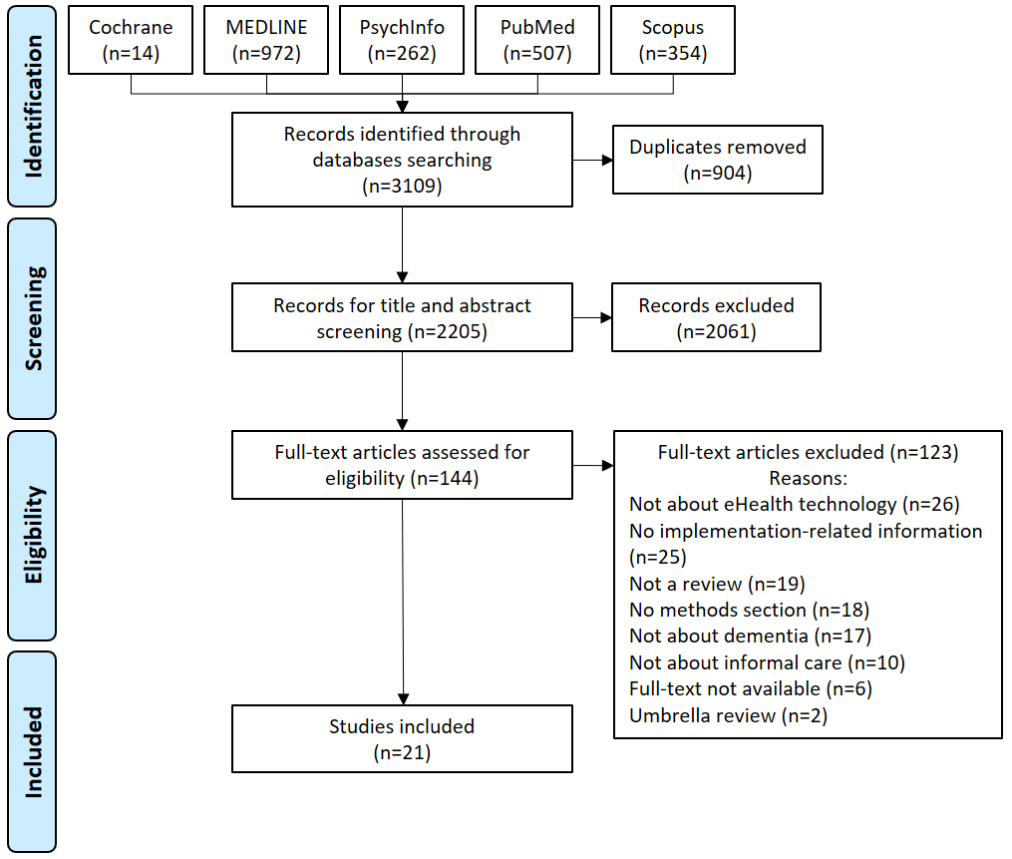
PRISMA (Preferred Reporting Items for Systematic Reviews and Meta-analyses) flow diagram of the study selection process.

Figure 1 illustrates the process of inclusion and exclusion of papers. The search strategy (available in Multimedia Appendix 1) produced 3109 results. After removing 904 duplicates and 2061 papers that were determined to be irrelevant according to our inclusion and exclusion criteria during title and abstract screening, 144 articles were considered for full-text screening. Finally, 21 papers were included for this review. Reasons for exclusions are detailed in Figure 1.

Table 1 summarizes details about the included studies such as author, year, title, type of review, and number of studies included per review. The studies were published between 2014 and 2020 and the majority were systematic reviews. The quality of the included studies was evaluated according to the Critical Appraisal Checklist for Systematic Reviews and Research Syntheses checklist [30], which ranged from 55% to 100% (for further details see Multimedia Appendix 2).

The included studies varied in their usage of implementation-related terminology and focus. For instance, 43% (9/21) of the included studies did explicitly use the term “implementation” in the text. Those that did not often used terminology related to certain subcomponents of implementation such as adoption, effectiveness, acceptability, or acceptance instead. Furthermore, although all studies contained information related to implementation, only 5 out of 21 reviews exclusively acknowledged implementation, or certain subcomponents of it, as the main focus of the study.

**Table 1:** Characteristics of the included studies.

Reference	Year	Title	Type of review	Number of included studies	Quality appraisal (%)
Armstrong and Alliance [31]	2019	Virtual support groups for informal caregivers of individuals with dementia: a scoping review	Scoping	25	100
Brando et al [32]	2017	The application of technologies in dementia diagnosis and intervention: A literature review	Literature	30	75
Christie et al [14]	2018	A systematic review on the implementation of eHealth interventions for informal caregivers of people with dementia	Systematic	46	95
Guisado-Fernández et al [22]	2019	Factors influencing the adoption of smart health technologies for people with dementia and their informal caregivers: scoping review and design framework	Scoping	109	100
Holthe et al [23]	2018	Usability and acceptability of technology for community-dwelling older adults with mild cognitive impairment and dementia: A systematic literature review	Systematic	29	95
Hopwood et al [24]	2018	Internet-based interventions aimed at supporting family caregivers of people with dementia: systematic review	Systematic	40	80
Hung et al [33]	2020	Using touchscreen tablets to support social connections and reduce responsive behaviors among people with dementia in care settings: A scoping review	Scoping	17	86
Klimova et al [34]	2019	E-learning as valuable caregivers’ support for people with dementia–A systematic review	Systematic	6	55
McKechnie et al [35]	2014	Effectiveness of computer-mediated interventions for informal carers of people with dementia—A systematic review	Systematic	14	100
Novitzky et al [36]	2015	A review of contemporary work on the ethics of ambient assisted living technologies for people with dementia	Literature	173	93
Rathnayake et al [37]	2019	mHealth applications as an educational and supportive resource for family carers of people with dementia: An integrative review	Integrative	7	93
Ruggiano et al [38]	2018	Rural dementia caregivers and technology: what is the evidence?	Systematic	30	100
Sanders and Scott [39]	2020	Literature review: Technological interventions and their impact on quality of life for people living with dementia	Literature	38	100
Sriram et al [40]	2019	Informal carers' experience of assistive technology use in dementia care at home: A systematic review	Systematic	56	100
Suijkerbuijk et al [18]	2019	Active involvement of people with dementia: a systematic review of studies developing supportive technologies	Systematic	49	75
Thordardottir et al [41]	2019	Acceptance and use of innovative assistive technologies among people with cognitive impairment and their caregivers: a systematic review	Systematic	30	100
Tyack and Camic [42]	2017	Touchscreen interventions and the well-being of people with dementia and caregivers: A systematic review	Systematic	16	85
Van Boekel et al [43]	2019	Perspectives of stakeholders on technology use in the care of community-living older adults with dementia: a systematic literature review	Systematic	46	90
Vermeer et al [44]	2019	What do we require from surveillance technology? A review of the needs of people with dementia and informal caregivers	Scoping	28	93
Waller et al [45]	2017	Computer and telephone delivered interventions to support caregivers of people with dementia: a systematic review of research output and quality	Systematic	34	100
Yousaf et al [46]	2019	Mobile-health applications for the efficient delivery of healthcare facility to people with dementia (people with dementia) and support to their carers: a survey	Literature	17	93

### Characteristics of Technologies to Support Informal Dementia Care

#### Overview

The following sections summarize the types of eHealth technologies that aim to support informal dementia care (either directly or indirectly) that have been studied in the included literature. More specifically, we present an overview of primary user groups and the purpose of identified technologies.

#### Primary User Groups of Identified Technologies

Based on the included reviews, we identified supportive technologies that aim to be used by different primary user groups, characterized by different levels of involvement of informal caregivers (from high to low). Based on Gibson et al [47], we arranged the technologies into three overall groups. Most of the identified technologies are designed to be used “by informal caregivers,” (mentioned in 17 out of 21 reviews), while fewer are meant to be used “by people with dementia” (mentioned in 9 out of 21 reviews) and “with people with dementia” (PwD and informal caregivers jointly; mentioned in 8 out of 21 reviews). This result also highlights that most of the technologies identified in this review entail high involvement of the informal caregivers.

#### Purposes of Identified Technologies Per User Group

**Figure 2:**
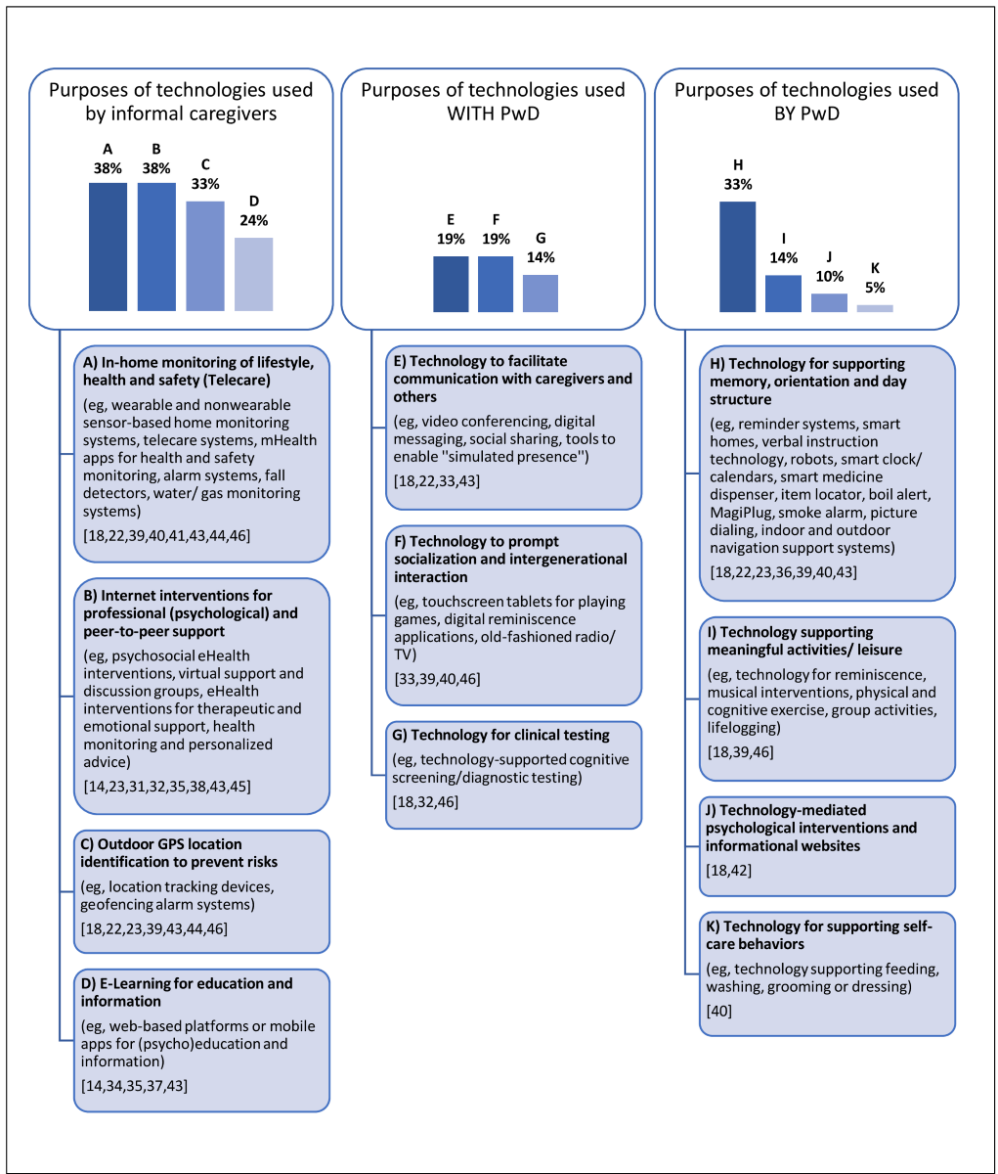
Purposes of technologies identified per primary user group. Percentages refer to the number of reviews mentioning a certain type and purpose of technology. One single review could mention different types and purposes of technologies.

As shown in Figure 2, overarching purposes of supportive technologies were identified within the different primary user groups.

Technologies used by informal caregivers were typically those operated without the active involvement of people with dementia, which were primarily specifically designed to support (in)formal caregivers. Within this user group, most of the included technologies were used for the purpose of in-home monitoring of lifestyle, health, and safety of people with dementia using wearable and nonwearable sensors, and internet-based interventions providing professional (psychological) support to caregivers (both represented within 38% of all reviews). The third largest type of technology, represented within 33% of the reviews, was outdoor GPS location identification to reduce risks by alerting caregivers. Lastly, internet-based platforms for electronic learning and information were identified in 24% of all reviews.

Technologies used with people with dementia usually included those that require active involvement of both people with dementia and caregivers, and/or establish a communication channel between people with dementia and their caregivers. Within this user group, most of the included technologies (represented within 19% of the reviews) were collaborative devices designed to facilitate communication between people with dementia and their informal caregivers or relevant others (eg, video conferencing). This type of technology also included tools to enable simulated communication or the “simulated presence” [33] of informal caregivers. Furthermore, we identified technologies to prompt socialization and intergenerational interaction such as touchscreen tablets for playing games or digital reminiscence applications in 19% of the reviews. Lastly, in a minority of reviews (14%), we identified technology for clinical testing.

Technologies that can be used independently by people with dementia usually included supportive devices that make everyday living easier, thereby indirectly creating relief for informal caregivers as well. Most of the represented technologies within the user group of people with dementia (highlighted in 33% of the reviews) included technology for supporting memory, orientation, and day structure such as reminder systems or smart medicine dispensers. Technologies supporting meaningful activities and leisure such as reminiscence or musical interventions were represented within 14% of the reviews. Finally, technology-mediated psychological interventions and informational websites (represented within 10% of the reviews) and technology supporting self-care behaviors such as feeding or washing were the least identified among all reviews.

### Factors Influencing Implementation

#### Overview

The following section presents identified factors (ie, potential barriers and facilitators) influencing the implementation of technologies to support informal dementia care. Implementation-related information collected across the reviews was grouped within the seven domains of the NASSS framework: condition, technology, value proposition, adopters, organization, wider system, and embedding and adaptation over time [28]. These domains refer respectively to the context of the specific health condition in which the technology is applied, characteristics of the technology, added value of the technology, factors related to adopters, characteristics of the implementing organization, and wider institutional and sociocultural context of policy and regulations. The last domain refers to the relation between the first six domains and the adaptation over time of the technology [28]. Textbox 1 presents an overview of identified factors influencing the implementation of technology to support informal dementia care, sorted by the corresponding NASSS framework domains and subdomains.

**Factors influencing implementation of technologies to support informal dementia care, structured according to the Nonadoption, Abandonment, Scale-Up, Spread, and Sustainability (NASSS) framework domains and subdomains. Factors that fall beyond the subdomains within “Condition” of the NASSS Framework are reported under “Miscellaneous”.**

**1. Condition**

Nature of condition or illness

- Deterioration of functional and cognitive resources [22]
- Increasing level of suspicion [22]
- Advantages and challenges of involving people with dementia in early-stage development [18,22,36,40,48]
- Timing and pace of technology introduction [14,18,22,39,40,43,44]
- Adaptation of technology along the disease progression [14,31,42,44]

Comorbidities, sociocultural influences

- Technology vs condition denial [22]

Miscellaneous

- Fear of breaking or losing the technology [43]
- Partnership between participant and researcher [18]

**2. Technology**

Material features

- Unobtrusive and familiar design (including physical appearance, simplicity, and usability) [22,23,36,39,42,44]
- Stigmatizing design [23,36,43]

Knowledge needed to use

- Technology literacy and access [14,22,24,31,36,41,44]

**3. Value proposition**

Demand-side value

- Perception of immediate advantage [41]
- Mismatch between expected and perceived benefits [22,41]
- Different or competing values of informal caregivers and patients [43]
- Lack of expected value through lack of personalization [36,40]

**4. Adopters**

Patients

- Reported within the domain “Condition” above

Informal caregivers

- Characteristics of informal caregiver that hinder or foster implementation (including motivation, digital literacy, training/education, attitude toward technology, perceived competence, ethnicity/culture, caregiving workload) [14,22,23,39-41,45]
- Expected or perceived technology burden (including privacy concerns, the fear of being replaced by machines, routine disturbance) [14,22,36,41]

**5. Organization**

Capacity to innovate

- Capacity for long-term technical user support [14,22,43]

Readiness for this technology

- Staff insecurity toward technology [14]

Nature of adoption/funding decision

- Resources for public relations [14]

Extent of change needed to routines

- Staff availability, replacement, and training [14]

**6. Wider system**

Political, policy

- Preference of health insurance companies for more classically delivered solutions [14]
- Local care policies [14]

Regulatory/legal

- Privacy and ethical issues [14,36]
- Interoperability issues [36]

**7. Embedding and adaptation over time**

Organizational resilience

- Monitoring intervention fidelity and active facilitation of the service uptake [14]

#### Challenges Related to the Condition

A high number of factors influencing the implementation of technology to support informal dementia care were found to be related to the condition of dementia. Among those factors, the *deterioration of functional and cognitive resources,* associated with progression of the disease, were reported to complicate the process of acquiring the new knowledge needed to use new technologies [22]. In addition, an *increasing level of suspicion* toward new things, the general disapproval of supportive technologies that remind patients of their own condition (especially for those in *condition denial* [22]), and the patients’ *fear of breaking or losing expensive equipment* [43] could act as additional barriers to implementation, related to the condition of dementia.

Furthermore, the *advantages and challenges of involving people with dementia in early-stage technology development* and the consequences for uptake were widely addressed in the reviews [14,18,22,36,40]. In terms of advantages, involving and codesigning with people with dementia promotes the realization that technologies are accessible and meaningful to them, and helps to make their needs explicit to the eyes of the developers [18], which in turn prevents technology abandonment and rejection [36]. However, involving people with dementia in the early stages of development when there is no concrete or physical prototype yet available creates challenges. For instance, people with dementia might experience difficulties in retrospection or anticipation of hypothetical/abstract scenarios [18], which are skills that are especially required in predesign phases. Using low-fi prototypes and letting people with dementia interact with them [18] could be one potential solution, although it would still require a certain degree of hypothetical thinking to imagine using the technology in everyday life situations [18]. Coherently, the reviews report that the extent of involvement by people with dementia in the development phase was moderate [18]: people with dementia were usually involved as informants, and most of the time during the postdesign evaluative stage [18]. In other stages, different stakeholders such as informal caregivers or experts were preferably involved [18].

Additionally, *the timing and pace of technology introduction* was widely considered as crucial [14,18,22,39,40,43,44]. Even though people with early-stage dementia have fewer difficulties in learning new things [18,39], they might not always see a benefit in using supportive technologies if introduced too early, as they often find themselves in denial about the severity of their condition or their need for help [22]. By contrast, when technologies are introduced at a later stage, it might be more difficult for people with dementia to adapt to them [43]. The pace of technology introduction is closely related to the feeling of familiarity with the device. A sudden introduction of technology could lead to rejection, especially in the case of wearable devices, where people with dementia could remove them if they seem unfamiliar, whereas caregivers often think their loved ones are going to accept technologies easily [44]. A more gradual introduction, making the technology almost invisible to the user [22], could potentially facilitate the adoption process and continuity of use.

Furthermore, certain technologies seem to be more suitable for different stages of disease progression, and it is important that they match the users’ level of skills [42]. Disregarding the stage of dementia has been reported as a barrier to uptake, even when it comes to technologies such as virtual support groups for caregivers [31]. Therefore, it might be particularly useful to create supportive technologies that are able to *adapt to the disease progression* [14,31,42,44]. In particular, the content of interventions needs to be fitting and up to date [14].

Lastly, to maintain and improve the involvement of people with dementia and prevent early dropout, establishing a proper *partnership between the participants and researchers* and keeping participants thoroughly informed about the research development are recommended [18].

#### Technology

Technology-related aspects influencing uptake largely centered around an *unobtrusive and familiar design*. In particular, technologies designed to be used by people with dementia should be intuitive and familiar and, if desired, mimic old technologies that people with dementia might already be acquainted with, thus eliciting recognition rather than recall [22]. They should have a uniform and coherent design in terms of fonts, colors, and the shape and size of buttons, and have a nonthreatening look [22]. The type of technology and its usability also plays an important role in the adoption/implementation process [23]. For instance, touchscreen technologies seem to be well-tolerated and, if well-designed, people with early-stage dementia require minimal training to use them [42]. Technologies to be used by people with dementia need to be especially simplified in design and appearance; even technologies that tell the time need to be as simple as possible, according to Sanders and Scott [39].

Furthermore, a *stigmatizing design* should be prevented as it often leads to rejection [36,43] by people with dementia and their caregivers. Devices can be stigmatizing both in terms of appearance, such as creating a “handicapped look,” and in terms of the signals they emit in public settings that might be embarrassing [36]. Devices should therefore match the user’s identity to be adopted and not be perceived as stigmatizing [23].

In addition to having *access* to an internet connection [24], *technology literacy* (ie, knowledge needed to use technology) was recognized as a determinant for implementation [14,22,24,31,36,41]. Hopwood et al [24] raised the matter of the “digital divide,” referring to the gap between those who can use internet-based technologies and those who cannot. Although younger caregivers may not have problems using digital technology, caregivers of people with dementia such as partners or spouses are more likely to be older themselves and might experience more difficulties [24]. Disregarding the digital divide often starts in an early stage of development, as a certain level of technology literacy is often an inclusion criterion to participate in research studies [24]. Reducing the complexity of digital technologies, supporting access with potential input from health professionals, and helping to understand the potential benefits that might come from using technology may aid in bridging the divide [22,24,41].

#### Value Proposition

For successful implementation of technology in informal dementia care, it must be clear for whom and how the technology generates value. In the included studies, only implementation-related factors related to the demand-side value (ie, the value to the user) were considered, whereas factors related to the supply-side value (eg, business cases and models, chances of return on investment, potential risks for investors) were largely underrepresented.

Thordardottir et al [41] highlighted the importance of how the benefits of a technology are communicated to people with dementia and their caregivers. The authors suggest that the *perception of an immediate advantage* is a key element of acceptance and value creation, which helps to prevent the abandonment of technology after a short period of usage, even when it comes to more obtrusive technologies.

Furthermore, the review of Guisado-Fernández et al [22] found that it was rather common for people with dementia and their informal caregivers to have unrealistic expectations of what supportive technologies might accomplish for them. Such a *mismatch between expected and perceived benefits* [41] can hinder technology adoption. Thordardottir et al [41] underline the importance of a correct matching between expectations of people with dementia and their caregivers before implementation and the actual benefits of the technology following the initial use. A mismatch in this regard can impede successful implementation as consequence of the users’ disappointment [41].

Moreover, although the expected or perceived benefits are usually in line with the original purpose of the technology, informal caregivers and people with dementia often perceive different features as valuable [43]. Insight into *different or competing values of informal caregivers and patients* is important to prevent contradictory perspectives from becoming a barrier to continue using a technology [43].

Lastly, as mentioned by Novitzky et al [36] and Guisado-Fernández et al [22], users often consider that a certain technology is not meant for them. This issue can occur when introducing “off-the-shelf’’ technologies, which lack *personalization* both toward people with dementia and their caregivers [40]. As Sriram et al [40] describe, many technologies needed to be customized to the individual situations of the carers and people with dementia, and abandonment was frequent when this was not the case.

#### Adopters

We found a broad range of factors influencing implementation relating to the adopters/primary users of technology to support informal dementia care. Factors related to people with dementia themselves and the context of their specific condition are summarized in the subsection ‘’Challenges Related to the Condition” above. We thus here report on identified factors related to the informal caregiver.

The largest group of factors that facilitate or impede implementation centered around *personal characteristics of the informal caregiver*. Among these, their motivation, digital literacy, and training and education were found to be important factors [14,23,39-41]. Their attitude toward technology may influence whether they begin to use technologies or interventions, and their perceived competence influences whether or not they continue to use the technology [22,45]. Relating to digital interventions, ethnicity and culture were frequently mentioned as influencing factors, suggesting a potential benefit to tailoring interventions to specific minorities before implementing them [14,40]. Furthermore, caregiving workload has been identified as an important factor regarding adherence to digital technologies: the busier informal caregivers were, the less usage took place [14].

Finally, factors related to *expected or perceived technology burden* such as privacy concerns of informal caregivers about using technology to document personal issues [14], the fear of being replaced by machines [22,36,41], and routine disturbance [22] have been reported to hinder implementation.

#### Organization

This section describes factors influencing implementation related to the implementing organization, namely the technology provider. Although these were among the least addressed factors within the included reviews, several factors could be identified.

Primarily, when distributing new technologies, the organization’s *capacity for long-term technical user support* plays a key role for sustainable implementation. Providers should have the capacity to deliver guidance to people with dementia and their caregivers on how to use the technology, allow sufficient time to practice, and provide face-to-face home assistance in case of technical glitches [22]. Software and content should be updated regularly. Related to this, the literature stresses the importance of sufficient *staff availability, replacement when staff leaves,* and regular *staff training* [14]. Certain staff characteristics such as *insecurity about technological or ethical issues* have also been reported to impede implementation [14]. Lastly, the lack of sufficient *resources for public relations*, which is a situation that mostly impacts smaller organizations, has been described as a barrier to sustainable implementation [14].

#### Wider System

As described by the NASSS framework [28], the wider institutional and sociocultural context is often key to explaining an organization’s failure or success in moving from a demonstration project to a fully spread and sustainable technology. Based on the reviews, we identified a limited number of factors that mainly relate to the context of policy and (legal) regulations.

In particular, the effect of *local care policies* has been described as an important factor [14]. In recent years, policy developments have increasingly recognized the possible benefits of innovative eHealth technologies. However, the constrained ability of health insurance authorities to support innovation and their *preference for more classically delivered care* have been identified as significant barriers to implementation [14].

Furthermore, the literature discussed certain *privacy and ethical issues* that can pose a barrier to implementation [14]. Novitzky et al [36] reported that caregivers of people with dementia are increasingly concerned about the ethical responsibility and legal liability for any possible misuse of a technology that is used in the home setting. For instance, Vermeer et al [44], who reviewed the literature on surveillance technologies in home-based dementia care, posed the question of who is authorized to know the location of the person with dementia and when the use of these types of technologies would or should result in legal issues.

Lastly, it has been reported that sustainable implementation of supportive technologies requires them to be developed in a way to ensure that they are *interoperable* with future systems [44].

#### Embedding and Adaptation Over Time

We found that aspects within this last domain were strongly underrepresented, with only one review reporting on aspects related to the continued evolution and adaptation of technology over time. In particular, the review of Christie et al [14] mentions some of the suggested long-term implementation strategies such as reconciling community and organizational characteristics, streamlining processes for *monitoring intervention fidelity*, and *active facilitation of the service uptake* [14].

### Identified Gaps

Table 2 presents an overview of the number of reviews describing factors related to implementation of technology supporting informal dementia care per the domains of the NASSS framework. Most of the reviews described factors relating to the technology and the condition of dementia, followed by reviews describing factors related to adopters (informal caregivers) and the technology value proposition. Factors relating to the implementing organization, the wider system, and embedding and adaptation of technology over time were the least represented. In conclusion, the most identified factors provide information about how the condition of dementia, the technology itself, its expected and perceived value (demand side), and the informal caregiver might influence successful implementation, whereas considerably less has been reported on factors relating to the implementing organization, the wider institutional and sociocultural of context of policy and regulations, and the continuing adaptation of technology over time.

**Table 2:** Number of reviews identified per domain of the Nonadoption, Abandonment, Scale-Up, Spread, and Sustainability (NASSS) framework (N=21)^a^.

NASSS framework domain	Number of reviews
Condition (people with dementia)	11
Technology	11
Adopters	8
Value proposition	5
Organization	3
Wider system	2
Embedding and adaptation over time	1

^a^Based on the information provided in a review, one single review could fall within multiple NASSS domains simultaneously. To prevent overlap between categories, factors related to people with dementia and their specific condition have been grouped under “Condition”; factors related to the informal caregiver are represented within the “Adopters” domain.

## Discussion

### Principal Results

Our study aimed at gaining a more complete understanding of why it is often difficult to implement eHealth technologies that have been specifically designed to support people with dementia and their informal caregivers in everyday life. According to Bauer and colleagues [49], the main goals of implementation science should be (1) identifying barriers and facilitators to the uptake of innovations and (2) developing and applying strategies to promote the successful implementation of innovations. Our umbrella review integrates knowledge that has been fragmented across different reviews until now by (1) providing an overview of the types of technologies that have been used and studied in the context of informal dementia care, and (2) exploring the factors influencing the implementation of those technologies.

Our review found that regardless of the difficulties that come with implementing supportive technologies, a broad range of existing or to-be-developed technologies are studied in the context of informal dementia care. Similar to Gibson et al [47], we generally identified technologies that aim to be used by different primary user groups, characterized by varying levels of involvement of informal caregivers, ranging from technologies used by informal caregivers to technologies used between caregivers and people with dementia to technologies used by people with dementia. It was possible to identify a certain degree of overlap between the categorization by user groups that emerged in our study and the structure of the NASSS framework, specifically with respect to how certain determinants of implementation refer to the patients (Condition and Adopters) and others to the caregivers (Adopters). However, we find the distinction between the domain of Condition and the subdomain “Patients” (embedded in the Adopter domain) to be less practical.

One of the largest groups of technologies found in our review were monitoring devices, including in-home monitoring of health and safety, and outdoor location identification of people with dementia, showing that this technology domain has developed rapidly and is seen as promising. We found that privacy and ethical issues were frequently mentioned as a barrier in relation to this type of technology; however, ways to overcome this barrier have mostly been unaddressed. In a previous study among potential users, we found that artificial intelligence–driven monitoring systems particularly require introduction in a way that prevents caregivers from feeling undervalued [50]. We have published a set of requirements that can benefit the development and introduction of in-home monitoring technologies aimed at supporting home-based dementia care [50].

An important finding of our study was the uneven distribution of references identified within the 7 domains of the NASSS framework. Although most reviews contained information on how the condition of dementia, the technology itself, its expected and perceived value, and the informal caregiver might influence successful implementation, considerably less has been reported on factors related to the implementing organization, wider institutional and sociocultural context, and continued adaptation of technology over time.

Interestingly, two included reviews came to a similar observation. The review from Christie et al [14], which focused on digital interventions for caregivers of people with dementia, found contextual factors related to implementing organizations and the wider context to be underrepresented in the included studies. Similarly, the implementation factors identified by Thordardottir et al [41] often related to a “micro level” (the individual user), whereas factors on the “meso level” (organizational processes) and “macro level” (national policy context) were less frequently found.

An additional blind spot that emerged from our study was the lack of information on factors related to the supply-side value, which was surprising as business modeling is crucial for the success of an eHealth technology and can serve on a strategic level to guide sustainable implementation [51].

Overall, there seems to be a mismatch between the focus of research performed on supportive technologies for people with dementia and their informal caregivers, and existing implementation frameworks. In our view, this might indicate (1) a misconception or partial mental model of implementation within researchers in the context of informal dementia care or (2) a lower interest in research about the wider contextual factors. In fact, researchers probably prefer to focus more on concrete, well-known, and measurable aspects of implementation instead of focusing more on abstract concepts. Nevertheless, these results could constitute a possible explanation to implementation failures that are very diffused in this (and other) contexts [52].

The identified mismatch between theory and research practice was also visible in the fact that most of the included reviews did not generally identify the use of implementation frameworks in their included studies nor did they employ such a framework to systematize the results. However, the latter could be explained by the fact that many of the reviews did not focus on implementation “as a whole” but rather focused on specific subcomponents of implementation such as acceptance or adoption. One review we included produced an ad hoc framework to guide the design of “smart health technology” [22]. Interestingly, this DemDesCon framework [22] also covers considerations to be made at the user, social, and development levels.

### Strengths and Limitations

To the best of our knowledge, this review is the first of its kind to explore factors influencing the implementation of eHealth technologies to support informal dementia care at this level of abstraction. By analyzing reviews (that included 840 studies in total) instead of primary studies, we were able to (indirectly) include a large knowledge base. According to the methodology employed, our results could lay the grounds to provide practical insights for decision making in the context of implementation of eHealth to support informal dementia care [30]. An additional strength of this review also lies in the rigor of the data extraction and analysis, with multiple researchers independently screening and analyzing the information.

However, some limitations must be considered. First, our results specifically refer to the context of informal dementia care and therefore are not necessarily generalizable to other implementation contexts. Second, due to the employed umbrella review design, we had to rely on the way review authors have summarized their findings. The level of detail provided within the included reviews varied; not all reviews provided a detailed description of the types and purposes of their included technologies, with some only providing a shorter summary. Lastly, although the authors kept close track of recent literature, due to technical time for finalization and publication of the manuscript, papers that would have otherwise been included might have been overlooked.

### Future Research

Our results suggest that more research is needed to understand how implementing organizations, the wider institutional and sociocultural context, and business modeling influence the successful implementation of technologies to support people with dementia and their caregivers, as many of the included reviews failed to address these aspects. In addition, future implementation research within this target group should increase its focus on continued adaptation and embedding of technology over time. As eHealth technologies to support informal dementia care develop rapidly, it seems essential for implementers not to fall behind the technological progress or eventual changes in context and care standards.

Lastly, our review provides an overview of factors influencing implementation, but it does not differentiate between different types of technologies in this regard. Future research should investigate what is needed for successful implementation of specific kinds of technologies to support people with dementia and their informal caregivers at home.

### Practical Recommendations

To help future developers in creating and successfully implementing meaningful technologies for both informal caregivers and people with dementia, we generally recommend use of the NASSS framework in combination with a holistic and iterative development approach, which views implementation not as a postdesign phase but rather intertwined with development right from the start. In light of our results, the CeHRes Roadmap [17,53]—a toolkit to guide holistic eHealth development—seems especially suitable in several ways. First, this roadmap pays special attention to the characteristics of and interrelation between relevant stakeholders, the (wider) context, and the technology. Second, it incorporates evidence-based methods from participatory development and human-centered design. Third, it focuses on cocreation of a business model even before a prototype of a technology is being made. In this way, possible implementation barriers such as those identified in our study can be addressed and accounted for at an early stage of development.

In addition, we present a synthesis of our most important results in the form of checklist (see Multimedia Appendix 3) aimed at promoting reflections and providing insights for readers interested in the field of technologies to support informal dementia care. The present checklist is intended for researchers, policymakers, practitioners, experts, and any other stakeholder interested in technologies for informal dementia care who want to gain specific insights on the implementation process and determinants. Specifically, readers are provided with (i) a concise overview of relevant aspects and domains of implementation identified in this review in light of the NASSS framework and (ii) best practices and recommendations. The first step to use this simple tool is to define the technology (or type of technology) that needs to be implemented, and its primary user group. Readers can make use of Figure 2 to navigate the different options. By filtering the most and least important domains, researchers can concentrate on the most relevant aspects. Moreover, the reader can rate their relevance, the extent to which they were addressed in design or implementation, and make use of the practical insights that directly derive from our review and CeHRes Roadmap [17,53].

### Conclusions

The increasing number of community-dwelling people with dementia worldwide underscores the need for innovative eHealth solutions that can provide support to both patients and their caregivers in the home setting. However, sustainable implementation of supportive technologies within this target group can be difficult. Our umbrella review has provided a comprehensive overview of eHealth technologies studied in the context of informal dementia care and contributes to a better understanding of a broad range of factors influencing their implementation. These findings can help researchers and stakeholders improve the development and implementation of eHealth technologies to support informal dementia care. More research is needed to identify the specific factors determining successful implementation related to the wider institutional and sociocultural context, the implementing organization and technology supplier, and continued adaptation and embedding of technology over time.

## Appendix

Multimedia Appendix 1 Search string.

Multimedia Appendix 2 Quality of the included studies.

Multimedia Appendix 3 Implementation of eHealth in the Informal Dementia Care setting: Practical Recommendations Checklist.

## Abbreviations

NASSS: Nonadoption, Abandonment, Scale-Up, Spread, and Sustainability
PRISMA: Preferred Reporting Items for Systematic Reviews and Meta-analyses
