# Supplementary material for: Factors Influencing Implementation of eHealth Technologies to Support Informal Dementia Care: Umbrella Review
Source: JMIR Aging. 2021 Oct 8;4(4):e30841. doi: 10.2196/30841 (PMC8538023; doi:10.2196/30841)
Supplement: Multimedia Appendix 1 [file aging_v4i4e30841_app1.docx]

**Search String Umbrella Review 03-06-2020**

| **Category** | **Search terms** |
| --- | --- |
| eHealth | (*technolog* OR ict OR it OR "smart solutions" OR ehealth OR e-health OR "electronic health" OR internet OR m-health OR *monitoring OR telehealth OR "computer-assisted" OR robot* OR “smart home*” OR “assisted living” OR system OR platform) |
| Implementation | (implement* OR success* OR fail* OR barrier* OR facilitat* OR evaluat* OR adopt* OR accept* OR *use) |
| Informal Care | ("informal care" OR "home care" OR carer* OR caregiv* OR “independent living” OR “living independent*” OR “community dwelling” OR family OR spouse OR partner) |
| Dementia | (dementia OR alzheimer* OR "cognitive impairment" OR "cognitive decline") |

Options and limits that were selected in the different databases:

| **Databases** | **Options and limits selected** |
| --- | --- |
| PubMed | - Results by year: 2010 - 2020 - Article type: Meta-Analysis + Review + Systematic Reviews + Scientific Integrity Review - Language: English, Dutch, German, Italian, Portuguese - Results: 507 |
| PsychInfo | - Limit to Publication date: 2010 – 2020 - Methodology: Literature review + Systematic review + Meta analysis + Metasynthesis - Language: English, Dutch, German, Portuguese (there was no Italian results) - Results: 262 |
| Cochrane Library | - Custom Year Range: 2010 – 2020 - No publication type nor language limit options were selectable - Results: 14 |
| Scopus | - Year: 2010 – 2020 - Document type: Review - Language: English, Dutch, German, Italian, Portuguese - Results: 1354 |
| MEDLINE (Web of Science) | - Timespan: 2010 – 2020 - Publication types: Review - Language: English, Dutch, German, Italian, Portuguese - Results: 972 |

Results (total): 3109 (PubMed 507, PsychInfo 262, Cochrane 14, Scopus 1354, Medline 972)

Duplicates removed automatically (Covidence): 904
