# Supplementary material for: Factors Influencing Implementation of eHealth Technologies to Support Informal Dementia Care: Umbrella Review
Source: JMIR Aging. 2021 Oct 8;4(4):e30841. doi: 10.2196/30841 (PMC8538023; doi:10.2196/30841)
Supplement: Multimedia Appendix 2 [file aging_v4i4e30841_app2.docx]

| Quality criteria  Author (Year) | 1 Is the review question clearly and explicitly stated? | 2 Were the inclusion criteria appropriate for the review question? | 3 Was the search strategy appropriate? | 4 Were the sources and resources used to search for studies adequate? | 5 Were the criteria for appraising studies appropriate?* | 6 Was critical appraisal conducted by two or more reviewers independently?* | 7 Were the methods used to combine studies appropriate? | 8 Was the likelihood of publication bias assessed?* | 9 Were recommendations for policy and/or practice supported by the reported data? | 10 Were the specific directives for new research appropriate? | Result |
| --- | --- | --- | --- | --- | --- | --- | --- | --- | --- | --- | --- |
| Armstrong (2019) | Met | Met | Met | Met | NA | NA | Met | NA | Met | Met | 7/7 (100%) |
| Brando (2017) | Met | Met | Met | Met | Met | Not met | Unclear | Not met | Met | Met | 7.5/10 (75%) |
| Christie (2018) | Met | Met | Met | Met | Met | Met | Met | Unclear | Met | Met | 9.5/10 (95%) |
| Guisado-Fernández (2019) | Met | Met | Met | Met | NA | NA | Met | NA | Met | Met | 7/7 (100%) |
| Holthe (2018) | Met | Met | Met | Met | Met | Met | Met | Unclear | Met | Met | 9.5/10 (95%) |
| Hopwood (2018) | Unclear | Met | Met | Met | Met | Met | Unclear | Not met | Met | Met | 8/10 (80%) |
| Hung (2020) | Met | Met | Met | Met | NA | NA | Met | NA | Unclear | Unclear | 6/7 (86%) |
| Klimova (2019) | Not met | Met | Met | Met | Not met | Unclear | Met | Not met | Unclear | Unclear | 5.5/10 (55%) |
| McKechnie (2014) | Met | Met | Met | Met | Met | Met | Met | Met | Met | Met | 10/10 (100%) |
| Novitzky (2015) | Met | Met | Met | Met | NA | NA | Met | NA | Met | Unclear | 6.5/7 (93%) |
| Rathnayake (2019) | Met | Met | Met | Met | NA | NA | Met | NA | Met | Unclear | 6.5/7 (93%) |
| Ruggiano (2018) | Met | Met | Met | Met | Met | Met | Met | Met | Met | Met | 10/10 (100%) |
| Sanders (2020) | Met | Met | Met | Met | NA | NA | Met | NA | Met | Met | 7/7 (100%) |
| Sriram (2019) | Met | Met | Met | Met | Met | Met | Met | Met | Met | Met | 10/10 (100%) |
| Suijkerbuijk (2019) | Met | Met | Met | Met | Met | Met | Met | Not met | Not met | Unclear | 7.5/10 (75%) |
| Thordardottir (2019) | Met | Met | Met | Met | Met | Met | Met | Met | Met | Met | 10/10 (100%) |
| Tyack (2017) | Not met | Met | Met | Met | Met | Met | Unclear | Met | Met | Met | 8.5/10 (85%) |
| Van Boekel (2019) | Met | Met | Met | Met | Met | Met | Met | Not met | Met | Met | 9/10 (90%) |
| Vermeer (2019) | Unclear | Met | Met | Met | NA | NA | Met | NA | Met | Met | 6.5/7 (93%) |
| Waller (2017) | Met | Met | Met | Met | Met | Met | Met | Met | Met | Met | 10/10 (100%) |
| Yousaf (2019) | Met | Met | Met | Met | NA | NA | Met | NA | Unclear | Met | 6.5/7 (93%) |

Points: Met (1), Unclear (0.5), Not met (0).

*Only applicable for systematic reviews.
