# Supplementary material for: Factors Influencing Implementation of eHealth Technologies to Support Informal Dementia Care: Umbrella Review
Source: JMIR Aging. 2021 Oct 8;4(4):e30841. doi: 10.2196/30841 (PMC8538023; doi:10.2196/30841)
Supplement: Multimedia Appendix 3 [file aging_v4i4e30841_app3.docx]

**Implementation of eHealth in the Informal Dementia Care setting: Practical Recommendations Checklist**

| **Type of technology** (e.g. Monitoring device/Peer to peer support online/communication technology etc.) | |  | | | |
| --- | --- | --- | --- | --- | --- |
| **User Group** (Informal caregiver/Person with Dementia/Both) | |  | | | |
| **Domains to consider** | | **Relevance** (R=Relevant. I= Irrelevant. U= unclear) | **Addressed** (Yes/No/Partially) | **Best Practices** | **Notes** |
| **Characteristics of the Condition of dementia** | |  | | | |
| Nature of condition or illness | Deterioration of functional and cognitive resources |  |  | Consider the extent to which the person with dementia must acquire new knowledge to use the technology. |  |
|  | Increasing level of suspicion |  |  | Integrate technology gradually and early on. Keep in mind a possible state of denial toward the condition. |  |
|  | Advantages and challenges of involving people with dementia in early-stage development |  |  | When possible, involve people with dementia in early-stage development. However, when codesigning together, keep in mind their possible struggle with retrospection/abstraction/hypothetical thinking. |  |
|  | Timing and pace of technology introduction |  |  | Introducing technology early on, keeping in mind that it could not seem relevant/necessary at the time.  Prefer a gradual rather than a sudden introduction |  |
|  | Adaptation of technology along the disease progression |  |  | When possible/applicable, prefer a technology or tool which adapts to the progression of the disease |  |
| Comorbidities, sociocultural influences | Technology vs. condition denial |  |  | Avoid designing technology that reminds patients of their own condition. |  |
| Miscellaneous | Fear of breaking or losing the technology |  |  | Provide reassurance and easy explanations on how to use the the technology. |  |
|  | Partnership between participant and researcher |  |  | Establish a proper relationship between participant and researcher and keep participants in the loop. |  |
| **Characteristics of the Technology** | | | | | |
| Material features | Unobtrusive and familiar design (including physical appearance, simplicity, and usability) |  |  | Avoid obtrsuive/loud /big/ stigmatizing design. Prefer a simplified design that mimics the appearance of familiar devices (e.g., clocks) *(CeHRes Roadmap-Persuasive Designs)* |  |
|  | Stigmatizing design |  |  |  |  |
| Knowledge needed to use | Technology literacy and access |  |  | Consider practical aspects of access to technology and required digital literacy. *(CeHRes Roadmap- Contextual Inquiry)* |  |
| **Value Proposition** | | | | | |
| Demand-side value | Perception of immediate advantage |  |  | Consider whether the technology creates immediate benefits and communicate those to users. |  |
|  | Mismatch between expected and perceived benefits |  |  | Promote realistic expectations of what the technology might accomplsh for users. |  |
|  | Different or competing values of informal caregivers and patients |  |  | Gain insight into who the technology creates vlaue for and cosider different or competing values between different stakeholders. |  |
|  | Lack of expected value through lack of personalization |  |  | Incorporate options for personalization when possible. |  |
| Supply-side value | Business model of developer |  |  | Create a clear business model in an early stage of development already which defines how the technology/ product creates, delivers and captures value. |  |
| **Adopters** | |  |  |  |  |
| Patients | Reported within domain ‘’Condition’’ |  |  |  |  |
| Informal caregivers | Characteristics informal caregiver that hinder or foster implementation (including motivation, digital literacy, training/ education, attitude towards technology, perceived competence, ethnicity/ culture, caregiving workload) |  |  | The informal caregivers are fundamental in the implementation process. Their attitudes and personal characteristics directly influence the success of the technology. Create a partnership and involve their needs and perspectives from the beginning. |  |
|  | Expected or perceived technology burden (including privacy concerns, the fear of being replaced by machines, routine disturbance) |  |  |  |  |
| **Organization** | | | | | |
| Capacity to innovate | Capacity for long-term technical user support |  |  | Organizations must remain available long after the kickoff of the technology to grant continuous support to their users. Furthermore, staff of the implementing organization should be sufficiently and continuously trained. *(CeHRes Roadmap- Operationalization)* |  |
| Readiness for this technology | Staff insecurity toward technology |  |  |  |  |
| Nature of adoption/ funding decision | Resources for PR |  |  |  |  |
| Extent of change needed to routines | Staff availability, replacement, and training |  |  |  |  |
| **Wider system** | | | | | |
| Political, policy | Preference of health insurance companies for more classically delivered solutions |  |  | Involve health insurance companies as stakeholder and consider their ability/ willingness to support innovation. |  |
|  | Local care policies |  |  | Consider the effect of local care policies in an early stage of development. |  |
| Regulatory/ Legal | Privacy and ethical issues |  |  | Gain insight into possible privacy and ethical issues in an early stage of development. |  |
|  | Interoperability issues |  |  | Consider the extent to which the technology is sustainable in terms of interoperability with future or existing systems. |  |
| **Embedding and adaptation over time** | | | | | |
| Organizational resilience | Monitoring intervention fidelity, and active facilitation of the service uptake |  |  | Work out long-term implementation strategies involving the facilitation of technology uptake and continous evaluation cycles (process and summative evaluations).  *(CeHRes Roadmap-Summative and Process Evaluation)* |  |

**Instruction to use:**

1. The first step to use this simple tool is defining the technology (or type of technology) that needs to be implemented, and its primary user group. Readers can make use of Figure 3 to navigate the different options. We imagine that the type of technology and user group would largely determine whether a (sub)domain is considered relevant in the specific case. E.g. when thinking of a WiFi monitoring device for detecting fall behavior (which does not require active participation of the PwD), the cognitive decline of the patient with dementia would have less impact on the implementation of the device, than it would have on a device that implies greater engagement in its employment. By filtering the most and least important domains, researchers can concentrate on most relevant aspects.
2. Once the target group and type of technology are set, the readers might consider the 7 domains originally proposed by the NASSS Framework, and the subdomains that were identified in this contribution. For each domain, different subdomains that were identified as determinants of (un)successful implementation in the present contribution are listed.
3. The reader can now easily rate their relevance, the extent to which they were addressed in design or implementation and make use of the practical insights that directly derive from our review.

CeHRes Roadmap Toolkit:

<https://www.utwente.nl/en/bms/ehealth/cehres-roadmap-toolkit/>

[This is a Multimedia Appendix to a full manuscript published in JMIR Aging. For full citation information see <http://dx.doi.org/10.2196/30841>.]
